# Prognostic value and functional consequences of cell cycle inhibitor p27^Kip1^ loss in medulloblastoma

**DOI:** 10.1186/2050-7771-1-14

**Published:** 2013-03-01

**Authors:** Beryl A Hatton, David W Ellison, Amar Gajjar, Marcel Kool, Matthew Fero, James M Olson

**Affiliations:** 1 Clinical Research Division, Fred Hutchinson Cancer Research Center, 1100 Fairview Avenue North, Mailstop D4-100, PO Box 19024, Seattle, WA 98109, USA; 2 Department of Pathology, St. Jude Children’s Research Hospital, 262 Danny Thomas Place, Memphis 38105, Tennessee; 3 Department of Oncology, St. Jude Children’s Research Hospital, 262 Danny Thomas Place, Memphis 38105, Tennessee; 4 Division of Molecular Genetics, German Cancer Research Center, Im Neuenheimer Feld 280, Heidelberg 69120, Germany; 5 Division of Pediatric Oncology, University of Washington/Children’s Hospital, 4800 Sand Point Way NE, Seattle, WA 98105, USA

**Keywords:** Medulloblastoma, p27^Kip1^, Sonic hedgehog, Cerebellum, Mouse cancer models, Prognostic factors

## Abstract

**Background:**

The cyclin-dependent kinase inhibitor p27^Kip1^ functions during normal cerebellar development and has demonstrated tumor suppressor functions in mouse models of medulloblastoma. Because P27 loss is associated with increased proliferation, we assessed whether P27 absence in surgical medulloblastoma specimens correlated with response to therapy in pediatric patients enrolled in two large studies. Additionally, we examined the functional consequence of *p27*^*Kip1*^ loss in the SmoA1 medulloblastoma model to distinguish whether p27^Kip1^ reduces tumor initiation or slows tumor progression.

**Findings:**

Analysis of 87 well-characterized patient samples identified a threshold of P27 staining at which significant P27 loss correlated with poor patient outcome. The same criteria, applied to a second test set of tissues from 141 patients showed no difference in survival between patients with minimal P27 staining and others, suggesting that P27 levels alone are not a sufficient prognostic indicator for identifying standard-risk patients that may fail standard therapy. These findings were in contrast to prior experiments completed using a mouse medulloblastoma model. Analysis of cerebellar tumor incidence in compound mutant mice carrying the activated *Smoothened* (*SmoA1*) allele that were heterozygous or nullizygous for *p27*^*Kip1*^ revealed that p27^Kip1^ loss did not alter the *frequency* of tumor initiation. Tumors haploinsufficient or nullizygous for *p27*^*Kip1*^ were, however, more invasive and displayed a higher proliferative index, suggesting p27^Kip1^ loss may contribute to SmoA1 medulloblastoma progression.

**Conclusions:**

These studies revealed P27 loss affects medulloblastoma progression rather than initiation and that this putative biomarker should not be used for stratifying children with medulloblastoma to risk-based therapeutic regimens.

## Introduction

Approximately 20% of medulloblastoma patients classified as standard risk fail therapy and succumb to their disease [1]. Current prognostic criteria stratify patients into standard- or high-risk groups based upon age, the extent of surgical resection and the presence or absence of disseminated disease [2]. The intensity of radio- and chemotherapy are determined by risk group, although significant impairments induced by current strategies are well established, particularly in younger patients [2,3]. Because of these devastating toxicities, efforts are underway to reduce therapy intensity for standard risk patients. As the pediatric neuro-oncology community begins to de-escalate standard risk therapy, it consequently becomes critically important to identify molecular markers that recognize children likely to fail therapy that would benefit from stratification to more intensive therapeutic regimens.

Studies in various mouse models have demonstrated that loss of a single *p27*^*Kip1*^ [GenBank: NM_009875] allele increases tumor formation, suggesting *p27*^*Kip1*^*’s* tumor suppressor function is haploinsufficient or acts in a dosage-dependent manner [4]. In the SmoA1 mouse medulloblastoma model, loss of P27 resulted in early death compared to counterparts with wildtype P27 levels [5]. While this work showed that P27 has tumor suppressive function in medulloblastoma, it remained to be determined whether loss of P27 caused an increase in tumor *initiation* or *progression*. This is an important distinction because molecules involved in tumor initiation are rarely good prognostic indicators, whereas a subset of molecules involved in tumor progression reliably predict response to therapy.

While *p27*^*Kip1*^mutations are generally rare, P27 abundance has provided a reliable prognostic marker for cancer progression in a subset of human cancers, such as breast, colon and prostate [6,7]. The prognostic value of P27 expression in human medulloblastoma is currently unknown. Previous studies using 14 human medulloblastoma samples found low or absent P27 expression in highly proliferative and undifferentiated tumor regions [8,9], but the sample size was too small to assess prognostic value.

This study first addresses the unanswered question regarding P27’s role in tumor initiation versus progression in experiments conducted in a genetically engineered mouse medulloblastoma model. This study additionally assesses P27’s potential as a prognostic indicator in children with medulloblastoma. The study evaluates whether clinical outcomes improve for children with high P27 first in a retrospective clinical trial cohort (training data set) and then in a larger clinical trial cohort (test data set).

## Methods

### Compound mutant mice

SmoA1 mice were crossed with heterozygous *p27*^*wt/-*^ mice to produce compound mutant (*SmoA1*, *p27*^*wt/-*^*and SmoA1, p27*^*−/−*^) mice. Mice were sacrificed at 2 months to examine tumor incidence via histopathology. Transgenic mice were maintained in accordance with the NIH Guide for the Care and Use of Experimental Animals with approval from our Institutional Animal Care and Use Committee.

### Immunohistochemical analyses

Mice were pulsed with BrdU (100 mg/kg) one hour prior to sacrifice. Tissues were paraffin embedded and cut into four micron sections. Sagittal sections were cut at multiple levels from each cerebellum analyzed and scored for the presence or absence of tumor formation. Tissue sections were stained with antibodies recognizing p27, cyclin D1 and BrdU, and five high power fields (HPF) were scored for each sample to quantify the proliferative index within cerebellar tumors. Human medulloblastoma tissues were stained with a monoclonal p27^Kip1^ antibody and detection carried out with 3,3’-diaminobenzidine reagent. Tissue microarrays containing tissues from 87 patient samples were previously described [10]. P27 protein expression levels were also examined on tissue microarrays containing tissues from 141 patients enrolled on the SIOP PNET03 (1992–2000) study [11,12]. Staining intensity was evaluated by observers blinded to patient and survival data using the Nikon Elements Imagining Software after slides were digitally scanned.

### Statistics

For mouse studies, sagittal sections were cut at multiple levels from each cerebellum analyzed to evaluate tumor incidence and a Fisher’s exact test was used to determine p values. A *t* test was used to determine p values for quantification of the proliferative index. For human patients, overall survival was calculated from diagnosis date until death or the last follow up date. Survival distribution was estimated by the Kaplan-Meier method and compared using the log-rank test (SPSS 15.0). A multivariate Cox proportional-hazards regression model, with overall survival as the dependent variable, was used to assess the impact of P27 expression levels on the three individual components of the current risk stratification model (metastasis, residual disease, age <3). Two-sided *p*-values of less than 0.05 using the 95% confidential interval were considered to indicate statistical significance.

## Findings

Genetic alterations have been isolated from medulloblastomas within signaling pathways that normally regulate proliferation and differentiation of granule neuron progenitors (GNPs) during cerebellar development [13]. Medulloblastomas are induced in the SmoA1 mouse model through the constitutive activation of the sonic hedgehog (Shh) [GenBank: NM_009170] pathway within GNPs, signaling normally required for GNP proliferation. *p27*^*Kip1*^ loss in mice can increase tumor formation [4], and *p27*^*Kip1*^ haploinsufficiency has been further supported by studies using the *Patched1* [GenBank: NM_008957] heterozygous (*Ptc1*^+/−^) and *SmoA1* mouse medulloblastoma models [5,14], where Shh-induced medulloblastoma incidence was accelerated by loss of one or both *p27*^*Kip1*^ alleles.

**Figure 1:**
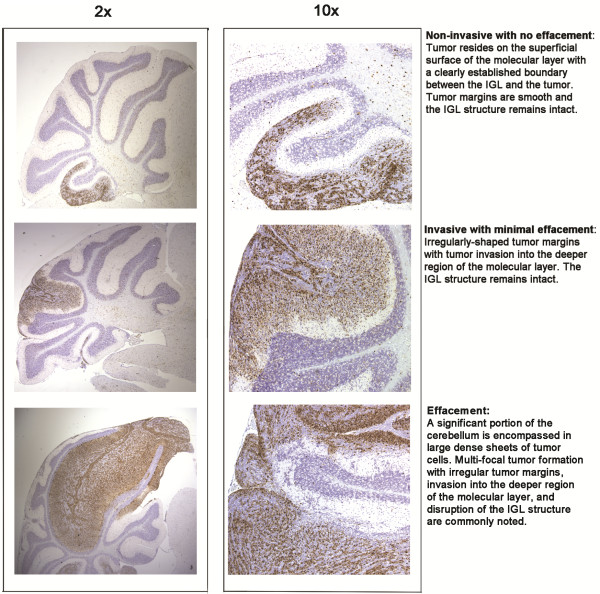
**Pathology criteria for SmoA1 tumor phenotype scoring.** Sagittal sections were cut at multiple levels from each cerebellum analyzed at the two-month time point and scored for the presence or absence of tumor formation. Sections displayed in the panels have been stained with a monoclonal cyclinD1 antibody to distinguish tumors (brown stain) from remaining normal cerebellum (blue Haematoxylin counter stain). Additionally, tumors were scored as being non-invasive with no effacement, invasive with minimal effacement or effaced according the criteria displayed in the panels above. Tumor pathology was graded by severity into one of the following three groups: (1) Non-invasive with no effacement: tumors reside on the superficial surface of the molecular layer with a clearly established boundary between the IGL and the tumor. Tumor margins are smooth and the IGL structure remains intact; (2) Invasive with minimal effacement: irregularly-shaped tumor margins with tumor invasion into the deeper region of the molecular layer. The IGL structure remains intact; and (3) Effacement: a significant portion of the cerebellum is encompassed in large dense sheets of tumor cells. Multi-focal tumor formation with irregular tumor margins, invasion into the deeper region of the molecular layer, and disruption of IGL structure are commonly noted.

**Figure 2:**
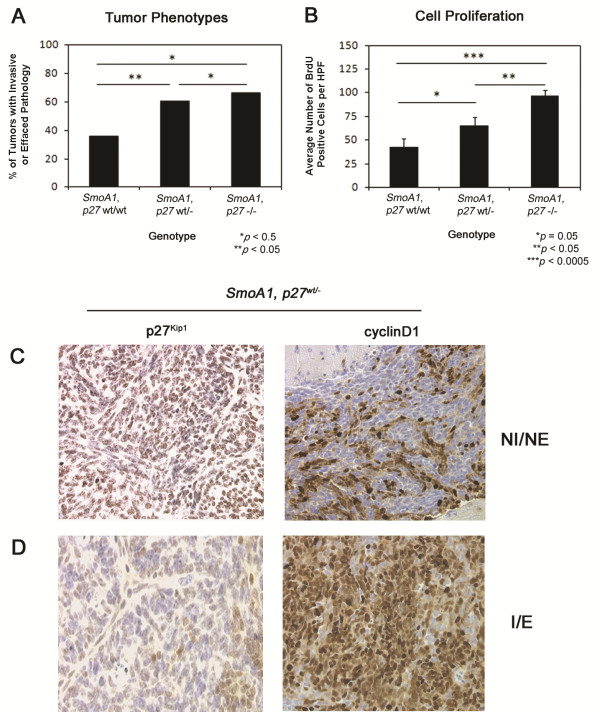
**Loss of *p27***^***Kip1 ***^**on the *SmoA1 *background facilitates tumor progression.** (**A**) *SmoA1, p27*^*wt/-*^ and *SmoA1, p27*^*−/−*^ mice demonstrated a significantly higher incidence of invasive and effaced tumors than *SmoA1, p27*^*wt/wt*^ littermates. A Fisher’s exact test was used to generate *p* values. (**B**) *SmoA1, p27*^*wt/-*^ and *SmoA1, p27*^*−/−*^ tumors demonstrated an increased proliferative index as determined by Bromodeoxyuridine (BrdU) uptake. Cerebellar sections were stained with an antibody recognizing BrdU and five high power fields (HPF) were scored for each sample. Bars represent the average number of BrdU positive cells per HPF, and error bars represent the standard error. A *t* test was used to determine *p* values. (**C**-**D**) Immunohistochemistry with monoclonal antibodies recognizing p27^Kip1^ and cyclinD1 demonstrated that tumor progression is associated with decreased p27^Kip1^ and increased cyclinD1 protein levels within tumors. Representative cerebellar sections from *SmoA1, p27*^*wt/-*^ cerebella showing p27^Kip1^ and cyclinD1 immunostaining in a non-invasive tumor with no effacement (NI/NE; **C**) and in a tumor with invasion and effacement (I/E; **D**).

To determine whether *p27*^*Kip1*^ loss affected medulloblastoma initiation or progression, we generated mice hemizygous for the *SmoA1* transgene that were heterozygous (*p27*^*wt/-*^) or nullizygous for *p27*^*Kip1*^ (*p27*^*−/−*^) and examined tumor incidence at two months of age, a time point preceding symptomatic medulloblastoma onset. Tumor incidence was 71.4% in *SmoA1*, *p27*^*wt/wt*^ mice (n = 35); 73.1% in *SmoA1*, *p27*^wt/-^ mice (n = 52); and 60% in *SmoA1*, *p27*^−/−^ mice (n = 20). The differences were not significant (*p* = 1.0 and *p* = 0.55, respectively). Tumors from heterozygous *SmoA1*, *p27*^*wt/-*^ mice did, however, display significantly more invasive phenotypes (Figure 1). 60.5% of *SmoA1*, *p27*^*wt/-*^ mice had invasive tumors and tumors with effacement compared to 36.0% of *SmoA1*, *p27*^*wt/wt*^ mice (Figure 2A, *p* = 0.04). Similarly, 66.7% of *SmoA1*, *p27*^−/−^ mice displayed invasive and effaced pathology (Figure 2A, *p* = 0.08). The proliferative index, measured by bromodeoxyuridine (BrdU) incorporation, was higher in tumors with invasive or effaced pathology and greater in advanced tumors from *SmoA1* mice lacking a single or both *p27*^*Kip1*^ alleles than in tumors from *SmoA1, p27*^*wt/wt*^ mice (Figure 2B, *p* = 0.05 and *p* = 0.0001, respectively). Increased tumor progression was associated with decreased p27^Kip1^ and increased cyclinD1 [GenBank: NM_009829] protein levels in tumors from both study groups (Figure 2C-D).

An important observation can be drawn from comparing tumor incidence at two months to the survival at later time point results reported previously. Early tumor formation was relatively unchanged by the loss of a single or of both *p27*^*Kip1*^ alleles in our study. In contrast, SmoA1 mice with wildtype *p27*^*Kip1*^ lived on average twice as long as mice with a single copy of *p27*^*Kip1*^[5], and mice retaining wildtype *p27*^*Kip1*^ in the heterozygous (*Ptc1*^+/−^) background survived significantly longer than counterparts lacking *p27*^*Kip1*^*,* which succumbed due to an increased tumor incidence [14]. In combination with the aggressive pathology observed in early tumors from *SmoA1* mice lacking *p27*^*Kip1*^, the higher tumor incidence in *Ptc1*^+/−^ mice lacking *p27*^*Kip1*^ further confirms that *p27*^*Kip1*^ is haploinsufficient in Shh-mediated medulloblastomas and that its loss of function contributes to medulloblastoma progression.

**Figure 3:**
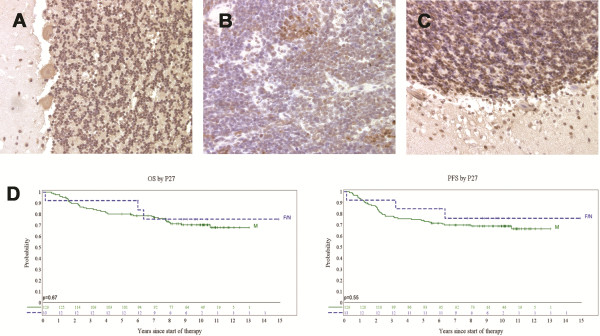
**P27 loss is a feature common in human medulloblastomas.** Human tumor tissue was obtained from the brain tumor tissue bank at Children’s Hospital in Seattle. These studies had prior approval from the Institutional Review Board and informed consent was obtained for all samples. Control brain samples were from four pediatric subjects (median age 16 yr, range 11-18 yrs) who died from trauma or non-malignant disease. These were from the Maryland Brain Bank, supplied by the laboratory of Dr. Gregory Riggins. (**A**) Representative P27 staining in the human cerebellum from a pediatric patient that died of non-cancer-related trauma demonstrates P27 expression in over 70% of granule cells. (**B-C**) Sections from human medulloblastoma samples were stained for P27, with panel (**B**) representative of the observed P27 protein loss within tumors. (**C**) Granule neurons within the remaining normal cerebellar architecture from the tumor sample shown in panel (**B**) exhibit strong, nuclear P27 staining. **(D)** Kaplan-Meier analysis from the second study of 141 patients enrolled on the SIOP PNET3 study [11,12]. Patients were grouped into three categories: N, with virtually no (<1%) P27-positive cells (n = 2), F, with less than 10% P27-positive cells and M, with greater than 10% P27-positive cells. Patients from groups F and N were grouped together because they represented only 13 of the 141 patients examined. Because desmoplastic/nodular tumors have a heterogeneous P27 staining pattern with pockets of strong P27 expression among fields of low or variable staining, we analyzed the test data set including and excluding patients with desmoplastic/nodular tumors, but neither analysis revealed prognostic value for P27.

We next assessed whether P27 levels could distinguish children likely to fail therapy from those likely to be long-term survivors. To properly evaluate P27 as a potential marker of therapeutic response, we established quantitative criteria for P27 staining in a training set of patient samples then applied the same criteria to a larger test set. Human medulloblastoma tissue microarrays containing tissues from 87 patient samples were analyzed for P27 expression [10]. 80% of samples evaluated had 20% or fewer P27 positive cells, with 16% of samples having less than 1% P27 positive cells. In contrast, intensity scoring of control sections from normal human cerebella revealed an average of 74% P27-positive granule cells (Figure 3A). Additional P27 staining on individual human medulloblastoma sections from five patients revealed significant loss of P27 protein (Figure 3B). Two tumor samples with regions lacking P27 contained additional regions resembling the remaining normal cerebellar architecture that stained positive for P27 (Figure 3C). Overall survival analysis demonstrated that patients with very low P27 expression had a poor outcome. Significant correlations were found when patients were stratified between high and low P27 levels, with patients with higher P27 levels (>1% P27 positive cells) having a more favorable outcome (n = 79, *p* = 0.027, data not shown). This correlation persisted when patients were additionally stratified by factors that define the standard-risk subgroup. P27 expression had prognostic value in patients greater than three years old with total surgical resection (n = 30, *p* = 0.001) or a lack of metastasis (n = 44, *p* = 0.007). While the number of patients with clinical data for all three standard-risk parameters was too small for Kaplan-Meier analysis, multivariate analysis with overall survival as the dependent variable demonstrated that P27 expression levels had prognostic value, independent of the extent of tumor resection or metastasis.

Together, these data suggested that P27 absence could further stratify standard-risk patients between survivors and those that fail therapy and do not survive. Because incorrect patient stratification could result in death for children placed improperly into a lower risk group, or unnecessary toxicity in children placed improperly into a higher risk group, we sought to confirm these findings by examining tissues from a second, larger study population. Tissues from 141 patient samples from children treated on the SIOP PNET03 clinical trial in Europe were stained for P27 and analyzed as above [11,12]. Only two patients were identified that met the <1% cutoff criteria, indicating that the threshold established in the training set was not suitable for the test data set. To determine if this threshold was too stringent, we relaxed the criteria to <10% to capture more patients with low P27 expression. No significant differences were found in overall survival (P = 0.67) or in progression free survival (P = 0.55) between patients with little to no (less than 10%, F/N) P27 cells and those with greater than 10% P27 positive cells (M) (Figure 3D). We therefore conclude that the first test set was a false positive signal, likely due to small numbers and retrospective establishment of the cutoff criteria. This discrepancy underscores the importance of evaluating potential prognostic indicators in large patient populations and using both training and test data sets when developing candidate prognostic indicators for clinical use. To our knowledge, this is the first quantitative analysis of P27 expression in a large cohort of human medulloblastoma samples.

## Abbreviations

GNP: Granule neuron progenitor; Shh: Sonic Hedgehog; IGL: Inner granule layer; BrdU: Bromodeoxyuridine; HPF: High power field; I/E: Invasive with effacement; NI/NE: Non-invasive tumor with no effacement.

## Competing interests

No authors of this paper have competing interests to disclose.

## Authors’ contributions

BAH conducted transgenic mouse studies and scored mouse pathology, immunohistochemically stained mouse and human tissue sections, participated in tissue imaging, data analysis and interpretation, and drafted and revised the manuscript. DWE, AG and MK provided tissue microarrays, assisted with data analysis and interpretation and assisted with manuscript revisions. MF provided transgenic animals, assisted with data interpretation and manuscript revisions. JMO provided transgenic animals, assisted with data interpretation and manuscript revisions. All authors read and approved the final manuscript.
